# Supplementary material for: A Transcriptomic Approach to Understand Patient Susceptibility to Pneumonia After Abdominal Surgery
Source: Ann Surg. 2023 Jul 27;279(3):510–20. doi: 10.1101/2023.01.25.23284914 (PMC10829899; doi:10.1101/2023.01.25.23284914)
Supplement: Supplementary file 1 [file sla-279-510-s001.docx]

**Supplementary figures:**

**Supplementary Figure Legends:**

**Supplementary Figure 1.** 

(A). Bar plot outlining the number of patients sampled for RNA sequencing at each time-point. (B). Principal component analysis of gene expression in samples preoperatively and postoperatively in 2-6, 24 and 48hrs. Each dot represents a sample, labelled with patient ID. Colours indicate different time-points. Shapes indicate the clinical status of patients postoperatively (circle: infection-free; circle plus: pneumonia). Solid arrow represents the trajectory of patient (ID: 049) later suffering pneumonia compared to the dashed line depicting a patient (ID: 047) remaining infection free. (C). Principal component analysis of gene expression in samples preoperatively and postoperatively in 2-6, 24 and 48hrs. Each dot represents a sample and is coloured with either sex (upper left panel), age (upper right), duration of surgery (lower left) or smoking status (lower right).

**Supplementary Figure 2.** 

(A). Cell counts (x10^9^/L) in samples across different time-points. *P* value was calculated by Wilcoxon signed-rank test.  * *P* < 0.05, *** *P* < 0.001, **** *P* < 0.0001,  *n.s.* = not significant. (B). Cell counts (x10^9^/L) in samples from patients who did (orange) or did not develop pneumonia (cyan) across different time-points. *P* value was calculated by Wilcoxon signed-rank test. *n.s.* = not significant. (C). Bar plot outlining the number of cancer patients who did or did not develop pneumonia. *P* values were calculated by two-tailed Fisher’s exact test. (D). Box plots of CIBERSORTx absolute scores of indicated cell types in samples from patients with (red) or without cancers (blue) across different time-points. *P* value was calculated by Wilcoxon signed-rank test. *n.s.* = not significant.

**Supplementary Figure 3.** 

(A-C). Heatmaps illustrate the enriched pathways/terms using gene set enrichment analysis. The top 5 enriched biological process GO terms (A), Canonical pathways corrected for cell proportions of neutrophils, monocytes and lymphocytes (B) or biological process GO terms corrected for cell proportions of neutrophils, monocytes and lymphocytes (C), in each contrast as indicated on the y axis were merged for visualisation.

**Supplementary Figure 4**. 

Dendrogram showing agglomerative hierarchical clusters in preoperative samples. The height on the y-axis represents the distance between two clusters. Two major clusters are highlighted in orange and cyan. The patient IDs together with the clinical status (1: pneumonia; 0: infection-free) are shown at the bottom of the dendrogram.

**Supplementary Figure 5.** 

Dot plots of the SRSq scores for samples from patients with (orange) or without (cyan) pneumonia. SRSq was quantified using the extended 19 gene signature to show robustness by predictive gene set.^18^ *P* value was determined using a linear model. 

**Supplementary Figure 6.** 

Predictive performance of ARISCAT score, a signature composed of 63 genes that were used to predict complex outcome in polytrauma patients,^29^ SRSq score and a signature composed of genes involved in neutrophil degranulation for detection of postoperative pneumonia in the BIONIC cohort. The prediction models for gene signatures and the combination of gene signatures with the clinical prediction model ARISCAT were constructed using a random forest approach as implemented in the R package caret (see **Supplementary Methods**). AUC and 95% CI was computed using the pROC package. AUC: Area under the (ROC) Curve.

**Supplementary methods:**

**Study details and ethics**

	The BIONIC (Biomarker based Identification Of Nosocomial Infective Complications) study was a prospective observational study. One hundred and fifty consecutive patients undergoing elective major abdominal surgery patients were recruited between April 2015 and August 2016 at the Royal London Hospital. Ethics approval was granted by the East Midlands – Nottingham 2 Research Ethics Committee (14/EM/1223).

**Inclusion and exclusion criteria**

	The inclusion criteria was age 45 or over, undergoing scheduled surgery involving the gastrointestinal tract, requiring a general anaesthetic and at least an overnight hospital stay 

	The exclusion criteria were refused consent and emergency surgery. Every patient on a weekday elective operating list was screened. Eligible patients were then approached for written, informed consent. Entry into the study did not influence clinical management. All patients received standardised perioperative prophylactic antibiotic therapy.

**Data Collection**

	Data were collected, in real time, on each patient until hospital discharge and included information on co-morbidities, American Society of Anesthesiology (ASA) physical status classification, indication for surgery, cancer staging and diagnosis, duration of the procedure, planned postoperative intensive care unit admission and in-hospital mortality. Risk of postoperative pulmonary complications were calculated using the Assess Respiratory Risk in Surgical Patients in Catalonia (ARISCAT) score,^1^ seen to be the best clinical discriminator in stratifying risk of postoperative pulmonary complications after major abdominal surgery.^2^

**Pneumonia diagnosis**

	Patients were examined daily for the presence of infection. Definitions of infection were agreed prospectively by the investigators and were based on the Centre for Disease Control and Prevention (CDC) criteria.^3^ The criteria for the diagnosis of pneumonia is outlined in **Supplementary Table 1**.  

**Sepsis diagnosis**

	Sepsis was diagnosed based on the Sepsis-3 criteria,^4^ with all patients also exhibiting two or more of the systemic inflammatory response syndrome (SIRS) criteria.^5^ Septic shock classified as hypotension requiring vasopressor therapy to maintain a mean arterial pressure of 65mmHg (or greater) and having a serum lactate level greater than 2 mmol/L after adequate fluid resuscitation.^6^

 

**Blood sampling**

	Blood samples were drawn immediately before induction of anaesthesia (preoperatively) and then between 2-6, and at 24 and 48hrs following the operation. Lack of availability of research staff impinged on the practicalities of obtaining the 2-6hr sample so sampling at this time was discontinued after the first 50 patients. PAXGene (Qiagen, USA) RNA tubes were collected and stored at -80^o^C until analysis. A paired EDTA sample was drawn alongside each PAXGene tube and a differential leukocyte count was performed by hospital laboratory staff, using a Sysmex SE2100 Analyser (Sysmex, Milton Keynes, UK).

**RNA extraction**

RNA was collected in PAXGene (Qiagen, USA) tubes and extracted using the PAXGene RNA extraction kit, with a DNase step to remove contaminating DNA. The integrity and quality of the total RNA were assessed on an Agilent 2100 Bioanalyzer (Agilent Technologies, Germany) by means of the RNA 6000 Nano Assay (Agilent Technologies, Germany). Globin and ribosomal RNA were removed via a Ribo-zero kit (Illumina, USA) and library preparation was carried out using the TruSeq Stranded Total RNA Library Prep Kit (Illumina, USA). Next generation sequencing was performed on the NovaSeq sequencing platform (Illumina, USA) at the Wellcome Centre for Human Genetics (WHG) in Oxford.

**RNA-Sequencing data analysis**	

	RNA sequencing read quality was assessed by FastQC (v0.11.9), with reads trimmed using Trim Galore (v0.6.2) and mapped to human genome primary assembly GRCh38 using STAR (v2.7.3a)^7^ in multi-sample 2-pass mode and based on the ENCODE recommended parameters. The aligned BAM files were quality checked via RNA-SeQC (v2.3.6), and then used to determine the gene counts through GENCODE annotations (release 34) and featureCounts (v1.6.4). After RNA-seq mapping quality assessment, one sample (ID:133 preop) with a low mapping rate and high proportions of duplicates was flagged as poor quality and removed from further analysis. Potential sample swaps were checked using CrosscheckFingerprints function from Picard (v 2.21.1).^8^ For gene differential expression analysis, the raw read counts were used as input into the R package DESeq2^9^ (v1.28.1) for pair-wise comparisons. Genes that had <10 reads mapped in >90% of the samples and the sex chromosome genes were filtered, retaining 18,653 genes for downstream analysis. Genes with fold change >1.5 and FDR <0.05, as per condition, were considered to be differentially expressed. 

RNA sequence data has been deposited at the European Genome-phenome Archive (EGA), under accession number EGAS00001007229; access is managed by a Data Access Committee.

All analyses were performed using the R software (v4.0.3).

**Validation datasets**

Trauma cohort:^10^ Files with the normalised gene expression data (microarray) and the clinical data were retrieved from [https://github.com/C4TS/HyperacutePhase](https://github.com/C4TS/HyperacutePhase)

Perioperative cohort:^11^ Files with the normalised gene expression data (microarray) and the clinical data (batch1) were retrieved from [https://data.mendeley.com/datasets/rhc5s6zj88/3](https://data.mendeley.com/datasets/rhc5s6zj88/3) 

**Comparison gene signature**

	A 63 gene score distinguishing patients with a complicated outcome following polytrauma^12^ was assessed in this cohort.

**Cell type deconvolution of bulk RNA-seq data**

	Cell-type deconvolution was performed with CIBERSORTx^13^ using a reference panel derived from a single cell RNA-seq cohort of 26 sepsis patients including 9 convalescent samples, 6 healthy donors and 7 post-cardiac bypass surgery patients.^14^ A signature matrix was built by the Create Signature Matrix analysis module with parameters min. expression = 0.25, replicates = 100 and sampling = 0.5. The CIBERSORTx^13^ absolute scores of each cell type in bulk samples were then obtained using the mixture file (Bulk RNAseq count matrix normalised by DESeq2),^9^ the signature matrix derived from single cell RNA-seq, the single cell reference matrix for S-mode batch correction and with 100 permutations via the Impute Cell Fractions analysis module.

**Gene enrichment and network analysis** 

The hypergeometric pathway enrichment analysis was performed as described previously.^15^ Differentially expressed genes, background genes expressed in this dataset (n=18,653), and the Reactome gene sets downloaded from Molecular Signatures Database (MSigDB; v2022.1) were used for analysis. Significance was determined using PHYPER function as implemented in R and multiple hypotheses testing by Benjamini–Hochberg correction. Gene-set enrichment analysis (GSEA) was performed using R package fgsea and MSigDB v7.4.1 including Canonical pathways (KEGG, BIOCARTA, REACTOME, PID and WikiPathways) and Gene Ontology (GO) terms. GSEA was carried out separately for each contrast, with genes ranked by both p-value and fold change/direction (-log_10_[*P-*value] x sign[log_2_FoldChange]). The top 5 enriched pathways in each contrast were selected for visualisation. GeneMania (v3.5.2)^16^ and Cytoscape (v3.9.0)^17^ were used to query and visualise the gene networks. 

**Clustering analysis**

Patient clusters were defined by hierarchical agglomerative clustering based on a similarity measure (Euclidean distance) and Ward's method or k-means (Hartigan-Wong algorithm). The R package factoextra (v1.0.7) was used for cluster visualisation.

**Machine learning**

We evaluated the performance of our gene signature with three repeats of 10-fold cross-validation and a random forest approach as implemented in the R package caret (v6.0.92). AUC and ROC curves of the models were calculated and plotted using the R package MLeval (v0.3).

**References**

1.	Canet J, Gallart L, Gomar C, et al. Prediction of postoperative pulmonary complications in a population-based surgical cohort. *Anesthesiology* 2010; 113(6):1338-50.

2.	STARSurg Collaborative, Tasman Collaborative. Evaluation of prognostic risk models for postoperative pulmonary complications in adult patients undergoing major abdominal surgery: a systematic review and international external validation cohort study. *Lancet Digit Health* 2022; 4(7):e520-e531.

3.	Horan TC, Andrus M, Dudeck MA. CDC/NHSN surveillance definition of health care-associated infection and criteria for specific types of infections in the acute care setting. *Am J Infect Control* 2008; 36(5):309-32.

4.	Singer M, Deutschman CS, Seymour CW, et al. The Third International Consensus Definitions for Sepsis and Septic Shock (Sepsis-3). *JAMA* 2016; 315(8):801-10.

5.	Levy MM, Fink MP, Marshall JC, et al. 2001 SCCM/ESICM/ACCP/ATS/SIS International Sepsis Definitions Conference. *Intensive Care Med* 2003; 29(4):530-8.

6.	Shankar-Hari M, Phillips GS, Levy ML, et al. Developing a New Definition and Assessing New Clinical Criteria for Septic Shock: For the Third International Consensus Definitions for Sepsis and Septic Shock (Sepsis-3). *JAMA* 2016; 315(8):775-87.

7.	Dobin A, Davis CA, Schlesinger F, et al. STAR: ultrafast universal RNA-seq aligner. *Bioinformatics* 2013; 29(1):15-21.

8.	Javed N, Farjoun Y, Fennell TJ, et al. Detecting sample swaps in diverse NGS data types using linkage disequilibrium. *Nat Commun* 2020; 11(1):3697.

9.	Love MI, Huber W, Anders S. Moderated estimation of fold change and dispersion for RNA-seq data with DESeq2. *Genome Biol* 2014; 15(12):550.

10.	Cabrera CP, Manson J, Shepherd JM, et al. Signatures of inflammation and impending multiple organ dysfunction in the hyperacute phase of trauma: A prospective cohort study. *PLoS Med* 2017; 14(7):e1002352.

11.	Lukaszewski RA, Jones HE, Gersuk VH, et al. Presymptomatic diagnosis of postoperative infection and sepsis using gene expression signatures. *Intensive Care Med* 2022; 48(9):1133-1143.

12.	Cuenca AG, Gentile LF, Lopez MC, et al. Development of a genomic metric that can be rapidly used to predict clinical outcome in severely injured trauma patients. *Crit Care Med* 2013; 41(5):1175-85.

13.	Newman AM, Steen CB, Liu CL, et al. Determining cell type abundance and expression from bulk tissues with digital cytometry. *Nat Biotechnol* 2019; 37(7):773-782.

14.	Kwok AJ, Allcock A, Ferreira RC, et al. Neutrophils and emergency granulopoiesis drive immune suppression and an extreme response endotype during sepsis. *Nat Immunol* 2023; 24(5):767-779.

15.	Zhang P, Kitchen-Smith I, Xiong L, et al. Germline and somatic genetic variants in the p53 pathway interact to affect cancer risk, progression, and drug response. *Cancer Res* 2021.

16.	Mostafavi S, Ray D, Warde-Farley D, et al. GeneMANIA: a real-time multiple association network integration algorithm for predicting gene function. *Genome Biol* 2008; 9 Suppl 1:S4.

17.	Shannon P, Markiel A, Ozier O, et al. Cytoscape: a software environment for integrated models of biomolecular interaction networks. *Genome Res* 2003; 13(11):2498-504.
