# Supplementary material for: A Transcriptomic Approach to Understand Patient Susceptibility to Pneumonia After Abdominal Surgery
Source: Ann Surg. 2023 Jul 27;279(3):510–20. doi: 10.1101/2023.01.25.23284914 (PMC10829899; doi:10.1101/2023.01.25.23284914)
Supplement: Supplementary file 2 [file sla-279-510-s002.docx]

| **Infection site** | **Definition** |
| --- | --- |
| **Pneumonia** | **Pneumonia** requires any 1 of the following: 1. Rales or dullness to percussion on physical examinations of chest **AND** any of the following: A. New onset of purulent sputum or change in character of sputum. B. Isolation of organism from blood culture. C. Isolation of pathogen from specimen obtained by trans-tracheal aspirate, bronchial brushing, or biopsy. 2. Chest radiography showing new or progressive infiltrate, consolidation, cavitation, or pleural effusion. **AND** any of the following: A. New onset of purulent sputum or change in character of sputum. B. Isolation of organism from blood culture. C. Isolation of pathogen from specimen obtained by trans-tracheal aspirate, bronchial brushing, or biopsy. D. Isolation of virus or detection of viral antigen in respiratory secretions. E. Diagnostic single antibody titre (IgM) or fourfold increase in paired serum samples (IgG) for pathogen. F. Histopathologic evidence of pneumonia. |

**Supplementary Table** **7.** Criteria for defining diagnosis of pneumonia
